# Immunogenicity and Efficacy of Pneumococcal Conjugate Vaccine (Prevenar13^®^) in Preventing Acquisition of Carriage of Pneumococcal Vaccine Serotypes in Tanzanian Children With HIV/AIDS

**DOI:** 10.3389/fimmu.2021.673392

**Published:** 2021-06-17

**Authors:** Geofrey Makenga, George Mtove, J. Kevin Yin, Abubakary Mziray, Veneranda M. Bwana, William Kisinza, Julius Mjema, Ben Amos, Laura Antony, Delane Shingadia, Shahin Oftadeh, Robert Booy

**Affiliations:** ^1^ National Institute for Medical Research (NIMR), Amani Research Center, Muheza, Tanzania; ^2^ Faculty of Medicine and Health, University of Sydney, Sydney, NSW, Australia; ^3^ National Centre for Immunisation Research and Surveillance, University of Sydney, Sydney, NSW, Australia; ^4^ St Augustine’s, Hospitali Teule, Private Bag, Tanga, Tanzania; ^5^ Great Ormond Street Hospital for Children NHS Trust, London, United Kingdom; ^6^ NSW and ACT Pneumococcal Reference Laboratory, Centre for Infectious Diseases and Microbiology, Institute of Clinical Pathology and Medical Research, Westmead Hospital, Westmead, NSW, Australia

**Keywords:** immunogenicity, efficacy, Prevenar 13, carriage acquisition, immunocompromised children, prevention

## Abstract

In every year, up to one million children die due to pneumococcal disease. Children infected with Human Immunodeficiency Virus (HIV) are mostly affected, as they appear to have higher rates of pneumococcal carriage and invasive disease. Successful immunity is dependent on mounting a sufficient immune response to the vaccine. We conducted a double blinded crossover randomised controlled trial to determine the serum antibody response (≥4-fold and geometric mean concentration) to pneumococcal vaccine (PCV13) serotypes at 3 months after second vaccination. We also determined the number and proportion of children carrying new (not present at baseline) vaccine serotypes of *S. pneumoniae* isolated from nasopharynx at 6 months post initial vaccination in recipients of Prevenar13^®^ compared with those given *Haemophilus influenzae*-type b (Hib) vaccine (control). The study was conducted at St Augustine's also known as Teule Hospital in Muheza, Tanga Tanzania. 225 HIV infected children aged 1-14 years were enrolled from Jan 2013 to Nov 2013 and randomised to Prevenar13^®^ or Hib vaccines each given at baseline and 2-3 months later. Nasopharyngeal and serum samples were collected at baseline and 4-6 months later. Serotyping was done by Quellung Reaction using Staten antisera. Serum antibodies were ELISA quantified. The study revealed a non-significant reduction in the acquisition of new vaccine serotypes of *S. pneumoniae* in the recipients of PCV13 by nearly a third compared to those who received Hib vaccine. The vaccine efficacy was 30.5% (95% confidence interval [CI] –6.4-54.6%, P = 0.100)]. The antibody response was not enough to induce a 4-fold rise in GMC in 7 of the 13 vaccine serotypes. When combining the effects of preventing new acquisition and clearing existing vaccine type carriage, the overall efficacy was 31.5% (95% CI 1.5-52.4%, P = 0.045). In the PCV13 group, the proportion of participants carrying vaccine serotype was significantly lower after 2 doses of PCV13 (30%; 32/107), compared with the baseline proportion (48%; 51/107). The introduction of PCV13 targeting HIV-positive children in a setting similar to Tanzania is likely to be associated with appreciable decrease in the acquisition and carriage of pneumococci, which is an important marker of the likely effect of the vaccine on pneumococcal disease.

**Clinical Trial Registration:**

https://www.anzctr.org.au/Trial/Registration/TrialReview.aspx?id=335579, identifier ACTRN12610000999033.

## Introduction

*Streptococcus pneumoniae* is a gram-positive bacterium that colonises the upper respiratory tract. It has long been recognised as a major cause of pneumonia, meningitis, septicaemia and other invasive infections (1). This bacterium exists in over 100 serotypes. The distribution of serotype-specific disease varies across populations (2).

The usual mode of transmission is person-to-person by respiratory droplet contact. *S. pneumoniae* infections are increased in both incidence and severity in persons with congenital or acquired humoral immunodeficiency including HIV disease (3–6).

Up to one million children die every year due to pneumococcal disease. These deaths are disproportionately represented in the developing world, particularly in children infected with Human Immunodeficiency Virus (HIV) (7). With an estimated 110,000 Tanzanian children living with HIV infection, *S. pneumoniae* is a significant cause of morbidity and mortality (8).

There is little information on the impact of pneumococcal vaccine in children older than those covered by the Expanded Programme on Immunisation (EPI) (9) especially in those infected with HIV. Successful protective immunity is dependent on mounting a sufficient antibody response to the vaccine. Colonisation is a pre-requisite for IPD (10), whereby, acquisition of a new serotype with high invasive potential typically precedes IPD. Children with HIV/AIDS have higher rates of both pneumococcal carriage as well as invasive pneumococcal disease (IPD) (7, 11). Thus understanding the impact of vaccination on colonisation is particularly important in this population.

Prevenar-13^®^ (PCV13) is a pneumococcal 13-valent conjugate vaccine covering serotypes 1, 3, 4, 5, 6A, 6B, 7F, 9V, 14, 18C, 19F, 19A and 23F which are ranked within the most common serotypes causing invasive disease in Africa. The vaccine has also been shown in multiple studies to be safe and effective (12–17).

We examined the efficacy of PCV13 in preventing the acquisition and carriage of its thirteen pneumococcal vaccine serotypes among Tanzanian children (aged 1-14 years) with HIV/AIDS using *Haemophilus influenzae*-type b (Hib) vaccine as the control. This study aimed to determine (1) the number and proportion of children carrying new (not present at baseline) vaccine serotypes of *S. pneumoniae* isolated from nasopharynx at about 2-3 months post 2^nd^ vaccination (given 2-3 months apart) in recipients of Prevenar13^®^ compared with those given Hib vaccine, and (2) the serum antibody response to pneumococcal vaccine serotypes at 2-3 months after the second vaccination. A secondary objective was to examine whether vaccine serotype colonisation reduced after vaccination.

## Materials and Methods

### Study Design

This was a double blinded crossover randomised controlled trial of the efficacy of 2 doses of Prevenar13^®^ (PCV13) in preventing acquisition of nasopharyngeal carriage of *S. pneumoniae* vaccine serotypes among HIV infected children aged 1-14 years. Eligible children were randomized to either Prevenar13^®^ or Hib vaccine. They received two doses of one of these vaccines intramuscularly 3 ± 1 months apart, however a single dose of Hib vaccine was given if there was evidence that a child had received three doses of Hib vaccine previously. Six months after the first dose and 3 ± 1 months later (i.e. at visit 3 and 4), enrolled children were given the first and second dose of the other vaccine respectively. Clinical assessment was conducted and a nasopharyngeal swab collected at baseline and at each follow up visit. Blood sample were collected at baseline and 6 ± 2months to measure antibody response to the vaccine. A total of 225 HIV infected children were enrolled from Jan 2013 to Nov 2013, each child was followed up for 12 months.

### Study Site and Population

The study was conducted at Muheza district, Tanga region in the North-eastern Tanzania between the foothills of Kilimanjaro and the East coast. Teule Hospital and its outpatient service a busy district-level general hospital, serving a surrounding population of 277,000. Teule Hospital houses the Diana Centre, one of the leading HIV treatment centres in the district. The Diana Centre was led by senior Tanzanian doctors and receives funding from government and charitable sources. A previous study looking at the nasopharyngeal carriage of pneumococcus in HIV-infected children and their caregivers was conducted at the same site (18). The Teule hospital was part of NetSPEAR (the Network for Surveillance of Pneumococcal Disease in the East African Region), which was a multicentre surveillance of the bacterial aetiology of severe childhood diseases (19). The laboratory and research staff has experience in processing nasopharyngeal swabs for bacterial culture. It should be noted that, by the time of the study commencement, Tanzania was still in the process of introducing PCV13 in the national EPI, and had not yet rolled out.

### Recruitment and Follow up

We screened and recruited HIV-positive children when they presented for routine follow-up to the HIV clinic at the Diana Centre. The use of anti-retroviral therapy did not influence eligibility. Participants meeting the following inclusion criteria could be enrolled: informed consent from the parent/guardian, HIV-positive, aged from 1year to 14years. We excluded individuals for the following reasons: refusal to participate, conditions contraindicating collection of a nasopharyngeal swab such as thrombocytopenia, contraindications to vaccination and previous vaccination with PCV13.

For children who were eligible for the study, their parent/guardian was provided an information sheet and consent form that were written in Kiswahili (local language). If they were unable to read, consent was explained to them in the presence of an impartial witness. After informed consent was obtained, we collected participants’ baseline data, such as presenting symptoms, HIV-history, socio-economic status and concomitant medications *via* interview and from the patient’s medical records.

**Figure 1:**
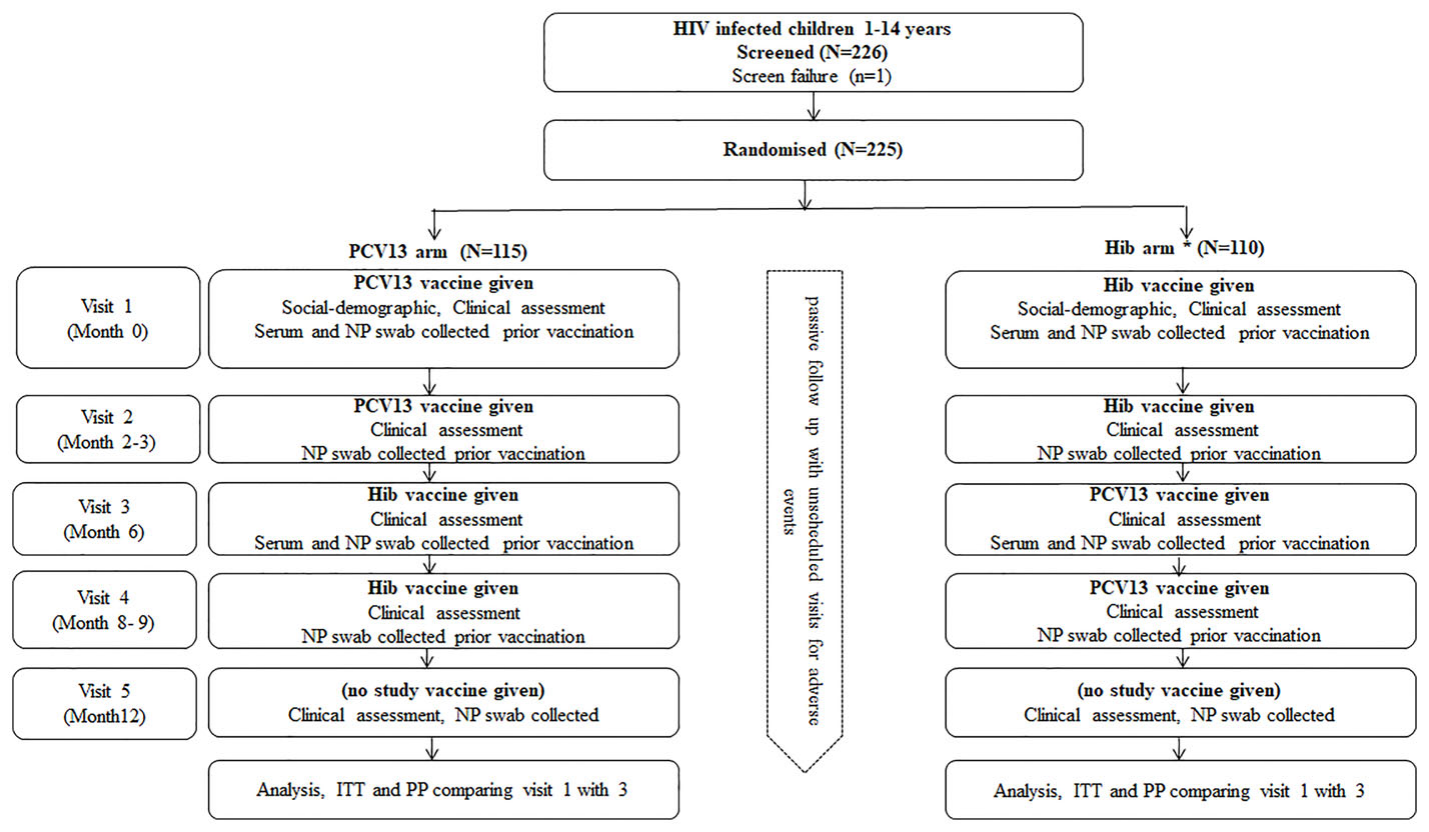
Study design flow chart. A single dose of Hib vaccine was given if there was evidence that the child had received three doses of Hib vaccine previously, HIV, Human immunodeficiency virus; PCV13, 13 valent Pneumococcal Conjugated Vaccine; Hib, Haemophilus influenza type b; NP, Nasopharyngeal; ITT, Intention to treat; PP, per protocol.

Eligible participants were randomised to receive either 2 doses of PCV13 or Hib conjugate vaccine given at baseline and between 2-4 months later (**Figure 1**). About 6 months after the first dose of vaccine, they received two doses of the other vaccine (shifted arms, PCV13 to Hib and Hib to PCV13) spaced 2-4months apart (i.e. at visit 3 and 4). There was a total of five study visits from recruitment to follow up as detailed on **Figure 1**.

Apart from the scheduled visit, participants were allowed to make unscheduled visit or call the study clinician in the event they fell sick or felt unwell. All adverse events were managed at the Teule hospital or could be referred to a regional referral hospital depending on the case. The reported adverse events were shared for review by the study Data and Safety Monitoring Board (DSMB).

### Randomisation and Blinding

Random numbers were generated *via* computer software in ‘excel’. The randomisation code was handled confidentially by the data manager. The random numbers were placed in brown security envelopes and stored in a file. For each subject, an envelope was randomly selected from the file. Depending on whether it contained an odd or an even number, the subject was assigned to receiving PCV or Hib respectively for the first two doses. The immunisation nurse handled the randomisation file confidentially. The immunisation nurse and the pharmacist were unblinded. The participant, investigators, and clinicians were blinded. Unblinding was done in the event of an adverse event that had to be reported to ethics, regulatory authority and the DSMB.

### Sample Collection and Processing

Nasopharyngeal and serum samples were collected according to the timeline specified in **Figure 1**. For swab taking, a paediatric calcium alginate tipped aluminium swab was inserted through the nose into the nasopharynx, and rotated gently through 180 degrees. Each swab was placed into 1ml of Skim milk tryptone-glucose-glycerin (STGG) transport medium, made according to WHO methods (20).

Nasopharyngeal samples were transmitted directly to the laboratory where they were cultured on 5µg/ml Gentamicin in sheep’s blood agar. 100µl of the sample were streaked onto the plate, which was incubated at 35°C in CO_2_ overnight, using a candle jar. Any resulting growth was sub-cultured onto blood agar by picking up to four individual colonies from the original plate and growing them for 24 hours at 35°C, with a disk of ethylhydrocupreine (optochin) for *S. pneumoniae* verification. Pneumococcal isolates were stored in both -30°C and -70°C freezers at St Augustine’s Hospital and later shipped to a pneumococcal reference laboratory at Westmead Hospital(Sydney, Australia) for serotyping by Quellung Reaction using Staten antisera and were all tested to find any mixed serotypes.

Sera were assayed at the Vaccine Research and Development (VRD) laboratory in Pearl River, NY in USA for antibodies to 13 individual pneumococcal capsular polysaccharides by the use of an ELISA (21). We did not employ the opsonophagocytic technique to assess whether the antibodies measured by ELISA were functional.

### Sample Size

The endpoint for the primary analysis was the presence of new (not present at baseline) vaccine serotype of *S. pneumoniae* isolated from participants in the PCV13 arm compared to those in Hib arm. To calculate the sample size needed, we endeavoured to predict the difference in proportions of subjects acquiring pneumococci (covered by the vaccine) between the two groups. We knew from our previous study at the same site that 81% of children were carrying *S. pneumoniae*, of which 49% of the isolated serotypes were vaccine types (18). So we estimated that in the control group 39.7% of subjects will be carrying vaccine serotypes (81% × 0.49=39.7). We assumed that, if the vaccine is 50% efficacious then we expected the study population to have a rate of 19.9% (39.7 × 0.5). In this way, we predicted that we would prevent acquisition in 19.9% of the treatment group. If the proportion of p1 (rate in control) was 0.3969 and the proportion for P2 (rate in treatment) was 0.1985, using a two sided test with an alpha level of.05 (P=.05) with power of 80% then sample size required was 91 per group. This worked out at a total number of 182 (91 x 2). Accounting for 20% dropout the final number was 223.

### Data Management and Statistical Analysis

Data was collected by both interview and from the patient’s medical records. Information were recorded in a standardised case report form (CRF). The CRF was designed to collect baseline data, such as presenting symptoms, HIV-history, socio-economic status and current medications. Height and weight were measured by standard methods using calibrated scales. Data were double entered in a Microsoft access database by two independent data clerks. A senior data manager reviewed the data regularly, compared between the two entries, and checked for completeness, accuracy, consistency and ranges. Data queries were resolved in consultation with the study clinician. Cleaned data were transferred into STATA version 12 for analysis. Data was analysed as per protocol. The main objective of the analysis was to evaluate the efficacy of PCV13 in preventing the acquisition and carriage of pneumococcal vaccine serotypes 3 ± 1 months after receipt of two doses. Vaccine efficacy against carriage was calculated as (1- RRv/u) X 100, where: RR (relative risk) v/u = Incidence ratio of the endpoint among children in the PCV group/Incidence rate of the endpoint among children in the Hib vaccine group. The endpoint for the primary analysis was the presences of new (not present at baseline) vaccine serotype of *S. pneumoniae* isolated from the nasopharynx. The endpoint for the secondary analyses was the serum antibody response (≥4-fold and geometric mean-fold) to pneumococcal serotypes 1, 3, 4, 5, 6A, 6B, 7F, 9V, 14, 18C, 19A, 19F, and 23F, 3 ± 1 months after the second dose. T-test was used to assess mean or proportional difference on various variables between children in the PCV13 group and those in the Hib vaccine group. We measured important confounders such as treatment with ARV, social economic status and various demographic data to check that our randomisation was adequate and adjusted if necessary.

### Study Agents and Vaccination

Each 0.5ml dose of Prevenar13^®^ (Wyeth/Pfizer) contains two micrograms of Pneumococcal polysaccharide serotypes 1, 3, 4, 5, 6A, 7F, 9V, 14, 18C, 19A, 19F, 23F and four micrograms of pneumococcal polysaccharide serotype 6B. It also contains sodium chloride and water for injection. The control agent, Hib (ActHIB^®^- Sanofi Pasteur) vaccine consists of the *Haemophilus influenzae* type b capsular polysaccharide (polyribosyl-ribitol-phosphate, PRP), covalently bound to tetanus toxoid. Both the lyophilised ActHIB vaccine powder and saline diluent contain no preservative. When reconstituted with saline, each single dose of 0.5 mL is formulated to contain 10 mcg of purified capsular polysaccharide conjugated to 24 mcg of inactivated tetanus toxoid, and 8.5% of sucrose. Each vaccine was administered by intramuscular injection provided in single-dose syringes. The vaccines were commercially labelled.

All vaccinations were administered according to specific product guidelines by a qualified immunisation nurse. The vaccination details were recorded on the participant’s clinic card as well as participant’s study CRF. As the clinic card remains stored at the Diana Centre, there was a backup to ensure that all children get both types of the vaccines at an appropriate schedule. Parents and guardians were shielded from seeing which vaccine was given.

### Ethical Consideration

The study was conducted in accordance with the declaration of Helsinki on ethical principles for medical research involving human subject adopted by Geneva assembly of the world medical association (22). In addition the study was conducted according to the approved protocol and the International Conference on Harmonisation- Good Clinical Practice (ICH-GCP) (23) and applicable local regulatory requirement to provide assurance that safety, rights, integrity, and confidentiality of trial subjects were protected and the reported results are credible and accurate. The study design enabled all participants to benefit from the two vaccines provided in the study. Access to both electronic and hard copy data was restricted to authorized study personnel only.

In Tanzania, the study obtained approvals from the National Health Research Ethics Committee (NatHREC) on 06 Sept 2012 with Ref No: NIMR/HQ/R.8a/Vol. IX/1392 and on 10^th^ Jan 2013 with Ref No. NIMR/HQ/R.8a/Vol. IX/1457. Also by the Tanzania Food and Drug Authority (TFDA currently known as TMDA) on 20 Jun 2013 Ref No. CE.57/180/05B/75. In Australia, the Westmead Children’s Hospital Sydney approved the study on 12 Jan 2011 and on 7 Jul 2012 under project number 10/CHW/62.

## Results

### Participants

A total of 226 participants were screened, and 225 were enrolled from Jan 2013 to Nov 2013. These were randomized to PCV13 (n=115) or Hib (n=110). Baseline socio-demographic and clinical characteristics were balanced between study arms and had no statistical significant difference between the two arms as shown in **Table 1**.

**Table 1:** Comparison of demographic and clinical characteristics of participants in two study arms.

Univariate analysis	All	PCV13 arm	Hib arm	% or mean difference (95% CI)	P-value
		(n = 115)	(n = 110)		
Age in years, mean (SD)	7.6 (3.5)	7.6 (3.3)	7.8 (3.7)	-0.2 (-1.1-0.7)	0.655
Female, n (%)	122 (54.2)	67 (58.3)	55 (50.0)	8.3 (-4.8-21.4)	0.215
Number of household members diagnosed with HIV, mean (SD)	0.9 (0.9)	1.0 (0.9)	0.9 (0.8)	0.1 (-0.1-0.3)	0.358
Children exposed to passive smoking in the house, n (%)	50 (22.2)	21 (18.3)	29 (26.4)	-8.1(-19.0-2.8)	0.145
Children sleeping in a room where cooking is done, n (%)	104 (46.2)	54 (47.0)	50 (45.5)	1.5 (-11.7-14.7)	0.822
Children currently attending school, n (%)	139 (62.1)	73 (64.0)	66 (60.0)	4.0 (-8.8-16.9)	0.536
Children with a recent (in the past week) respiratory infection, n (%)	96 (42.9)	46 (40.4)	50 (45.5)	-5.1 (-18.2-8.0)	0.443
CD4 count, mean (SD)	935.3 (559.6)	898.8 (515.5)	971.0 (600.2)	-72.1 (-234.2-89.9)	0.381
Children on Cotrimoxazole n (%)	160 (74.1)	83 (74.8)	77 (73.3)	1.4 (-20.4-13.3)	0.810
Children with signs of malnutrition, n (%)	15 (6.7)	11 (9.6)	4 (3.6)	5.9 (-0.6-12.5)	0.075
Children on Antiretroviral therapy (ARV), n (%)	190 (84.8)	98 (86.0)	92 (83.6)	2.3 (-7.2-11.8)	0.629
Number of household members, mean (SD)	5.2 (2.3)	5.0 (2.2)	5.4 (2.5)	-0.4 (-1.0-0.2)	0.203
Children whose caregiver had respiratory symptoms in past week, n (%)	75 (33.6)	32 (27.8)	43 (39.8)	-12.0 (-24.4-4.4)	0.059
Baseline Weight, mean (SD)	21.2 (8.3)	20.9 (7.4)	21.5 (9.1)	-0.6 (-2.8-1.6)	0.608
HIV clinical stage, median	3	3	3	NA	NA

HIV, Human immunodeficiency virus; PCV13, 13 valent Pneumococcal Conjugated Vaccine; Hib, Haemophilus influenza type b; CD4, cluster of differentiation 4; SD, Standard deviation; CI, Confidence interval.

Baseline pneumococcal isolation by culture was 71%, isolation continued with minimal decline throughout the study visits (**Table 2**). Total number of subjects who were lost to follow up during the study period was 33 (14.7%), this is less than the estimated dropout rate. The reasons for the loss to follow-up were death (8 cases), shifting to another region, going to school, and travelling following family conflicts.

**Table 2:** Enrolment (visit 1) and follow up (visits 2-5) status and proportion of participants with pneumococcal isolates by culture per visit.

Type of visit	Screening and Enrolment (N)	Swabs collected (N)	% Pneumococcal isolation by culture	Lost to follow up (N)
Screened	226	–	–	–
Visit 1	225	225	71.1	–
Visit 2	214	214	70.9	12
Visit 3	211	211	62.6	15
Visit 4	207	204	66.2	19
Visit 5	192	192	61.3	33

### Carriage

**Figure 2:**
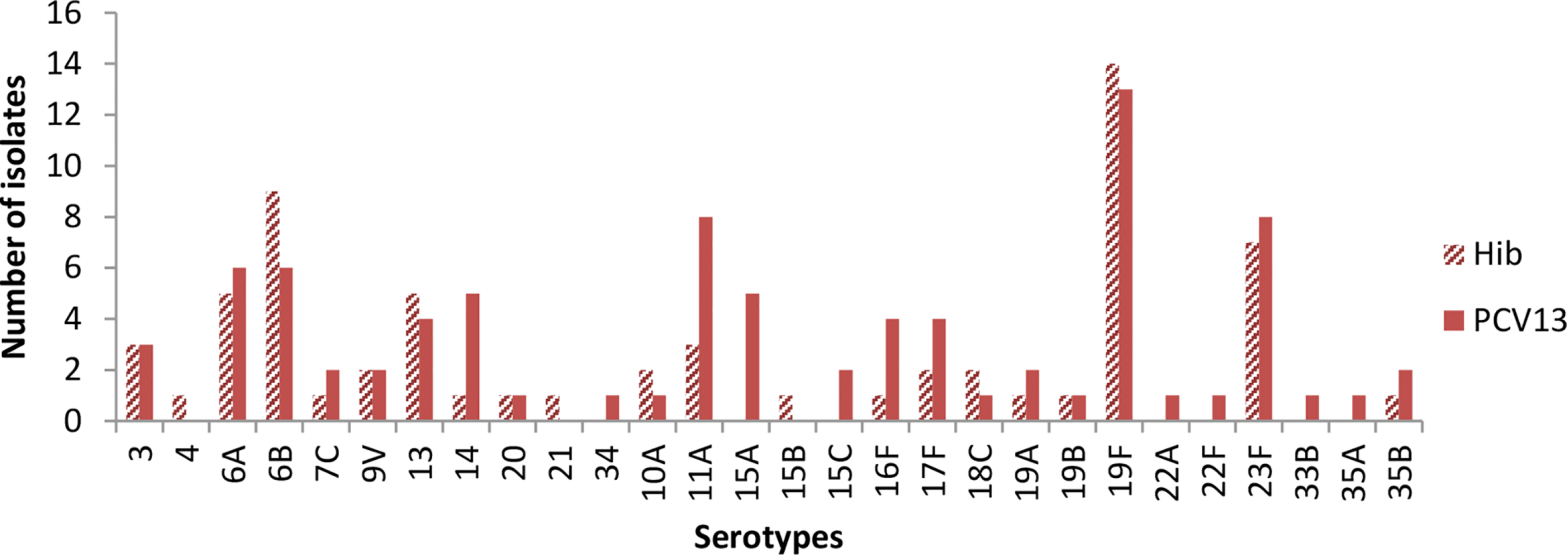
Pneumococcal serotypes isolated at baseline.

**Figure 2** shows baseline carriage of *S. pneumoniae* serotypes prior to vaccination. The three most common vaccine serotypes isolated in the Hib arm were 19F (n=14), 6 B (n=9) and, 23 F (n=8), while in the PCV13 arm, the three most commonly isolated serotypes were 19F (n=13), 11A (n=8) and 23F (n=8).

**Figure 3:**
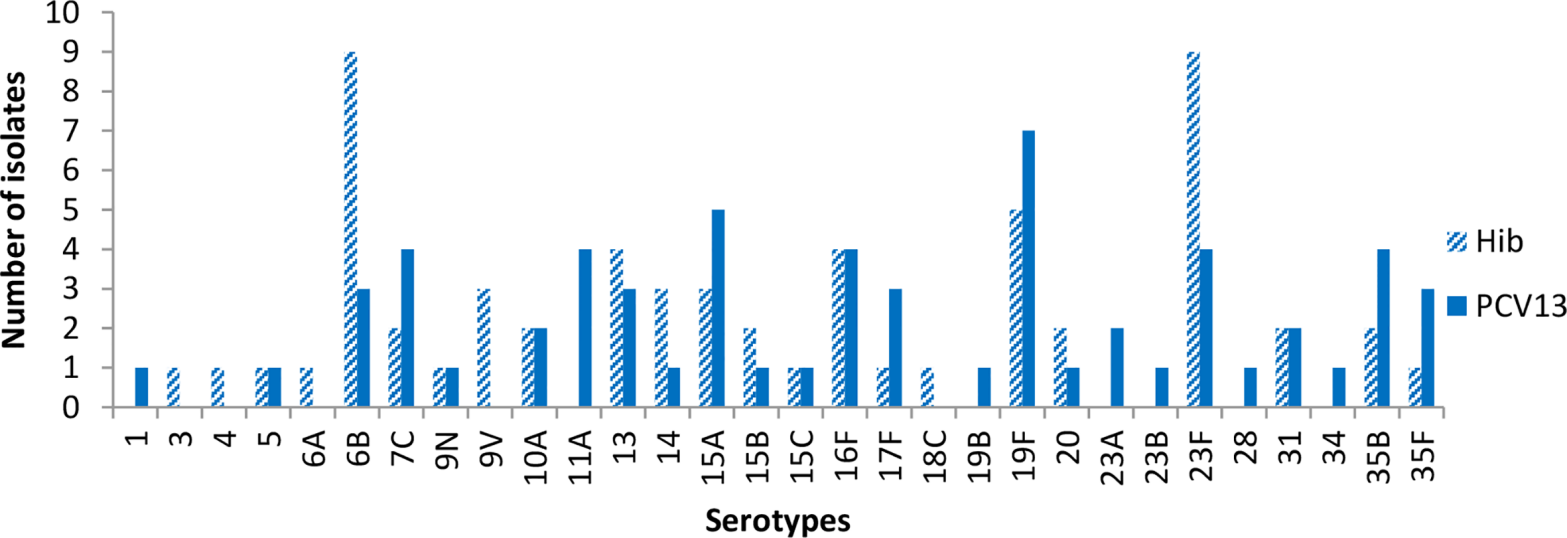
Pneumococcal serotype isolated at 4-6 months post baseline.

**Figure 3** shows carriage of *S. pneumoniae* at visit 3 (2-4 months post second dose of vaccine). The most common serotypes were 6B, 23F and 19F among individuals in the Hib group. In the PCV13 group, the most common isolates were 19F and 15A, while 5 serotypes were equally common at the third tier, 7C, 11A, 16F, 23F, and 35B.

### Efficacy Assessments

In our study, the efficacy of 2 doses of PCV13 on reducing acquisition of all vaccine serotype (primary study objective) was 30.5% (95% confidence interval [CI] –6.4-54.6%), which was just not significant (**Table 3**). When combining the effects of preventing new acquisition and clearing existing vaccine type carriage (secondary objective), the efficacy was significant at 31.5% (95% CI 1.5-52.4%) (**Table 3**).

**Table 3:** Within and between group comparisons of pneumococcal vaccine serotype carriage.

		Visit 1	Visit 3	
		N of participants carryingvaccine serotypes	N of participants developing new acquisition of vaccine serotype	N of participants carrying vaccine serotypes*
**Vaccine**	**PCV13**	51/107	26/107	32/107
	**Hib**	53/103	36/103	45/103
***p* value (2-sided) by column**		0.679	0.100	0.045
**Efficacy**		–	30.5% (–6.4-54.6%)	31.5% (1.5-52.4%)

*This column examines the combined effect in preventing the acquisition of (Visit 3) and clearing the carriage of vaccine serotypes (Visit 1).

On excluding Serotype 3 but including Serotype 6, PCV13 efficacy on preventing new acquisition of vaccine serotype was 32.1% (95% CI –6.3-56.6%; 24/107 vs. 34/103), and it was also just not significant. In addition, as expected, there was no significant effect after 2 doses of PCV13 on preventing new acquisition of non-PCV13 serotypes between the two groups (42/107 vs. 33/103; p=0.310).

Of note, in the PCV13 group, the proportion of participants carrying vaccine serotype was significantly lower after 2 doses of PCV13 (30%; 32/107), compared with the baseline proportion (48%; 51/107); p=0.011.

### Immunogenicity Assessments

**Figure 4:**
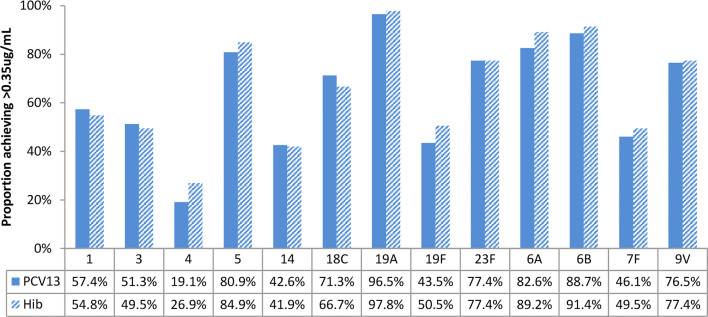
Baseline proportions of participants’ more than putative protective antibody concentration (0.35ug/mL), by study arms. No statistically significant differences were found for all vaccine serotypes.

**Figure 5:**
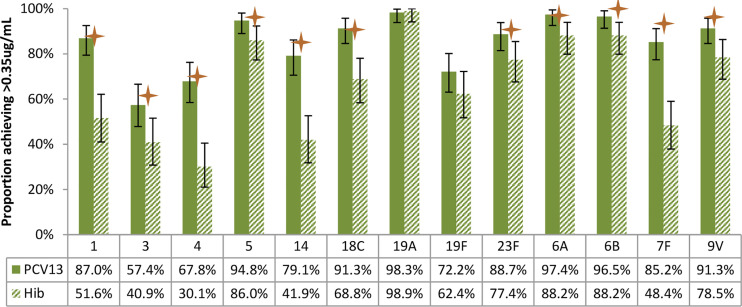
Post-vaccination (about 3 months after 2 doses) proportions of participants with more than putative protective antibody concentration (0.35ug/mL), by study arm. Statistically higher proportion of participants achieving putative protective antibody concentration in PCV13 vaccinees for 11 of the 13 vaccine serotypes (1, 3, 4, 5, 14, 18C, 23F, 6A, 6B, 7F, 9V) (star symbols over bars indicate statistical significance).

**Figure 6:**
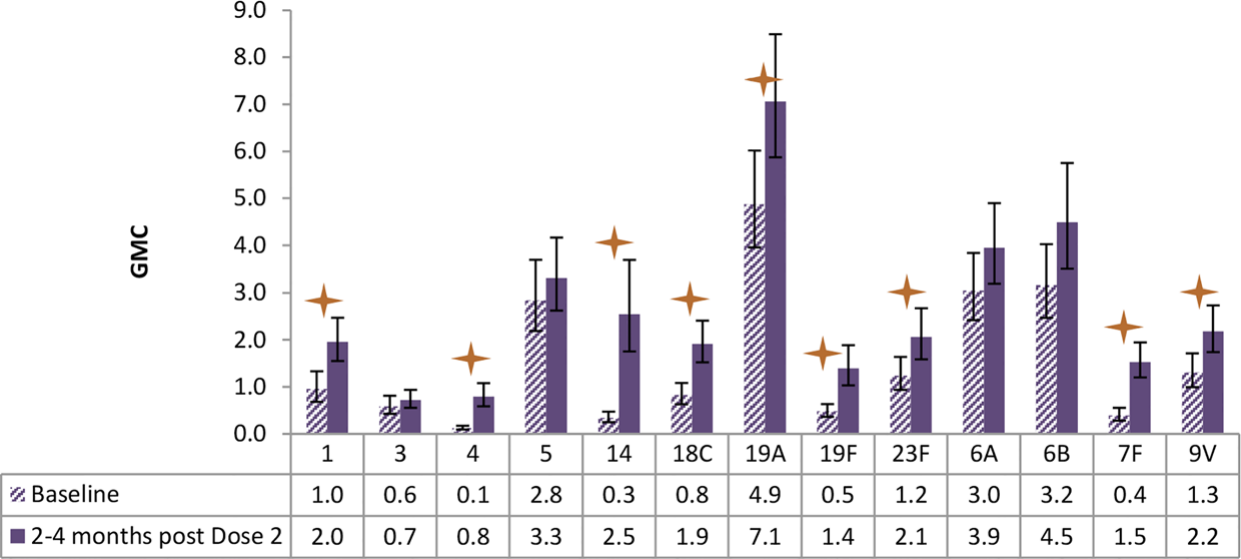
Antibody geometric mean concentrations (GMCs) at baseline and 2-4 months after Dose 2 of PCV13, by serotypes. Statistically higher GMCs were found in 9 of the 13 vaccine serotypes (1, 4, 14, 18C, 19A, 19F, 23F, 7F, 9V) (star symbols over bars indicate statistical significance).

**Figure 7:**
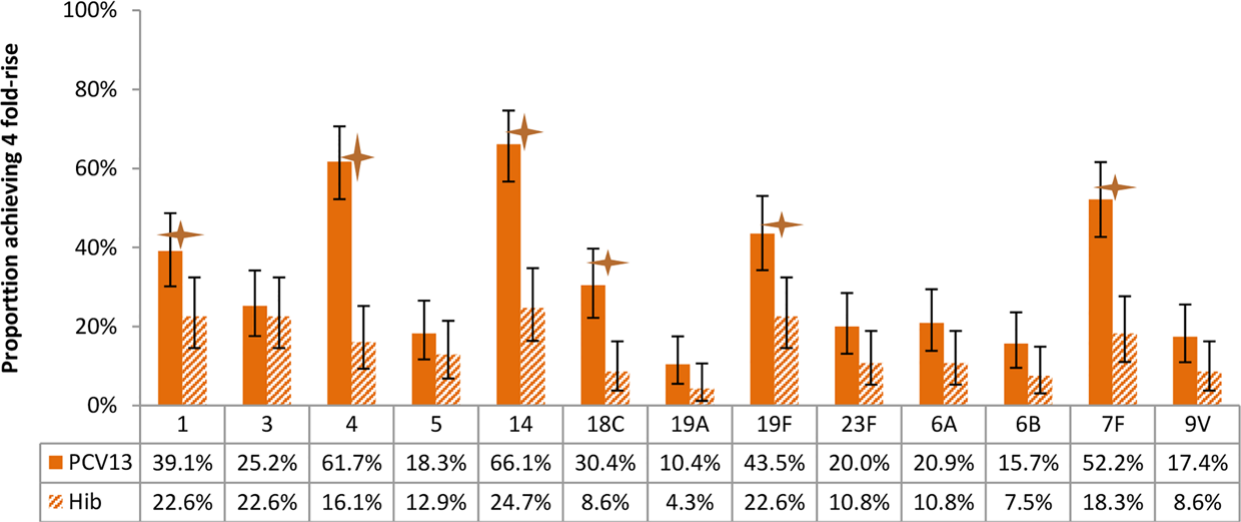
Proportion of participants achieving 4-fold rise of antibody concentration after vaccination, by arms. Statistically higher proportion of participants with 4-fold rise of antibody was found in PCV13 vaccinees for 6 of the 13 vaccine serotypes (1, 4, 14, 18C, 19F, 7F) (star symbols over bars indicate statistical significance).

**Figures 4**–**7** present the results of immunogenicity assessment by study arms. The baseline proportions of participants with more than putative protective antibody concentration (0.35ug/mL) were similar among participants of the two study arms (**Figure 4**). About three months post the second dose of vaccine; the proportions of participants with more than putative protective antibody concentration in the PCV13 arm were statistically significantly higher than those of the Hib arm for 11 of the 13 vaccine serotypes (**Figure 5**).

As shown in **Figure 6**, children receiving PCV13 developed statistically higher antibody GMC for 9 of the 13 serotypes as measured at about 3 months post the second dose of vaccination, in comparison of the GMC levels at baseline.

With respect to the proportion of participants achieving 4 fold-rise of antibody concentration after vaccination, statistically significant differences were found between study arms for 6 of the 13 vaccine serotypes (**Figures 7**).

### Safety Information

PCV13 was generally well tolerated by the participants. There was no statistical difference between study arms for solicited adverse events (AE), either local (such as injection site erythema or induration or injection site pain) or systemic (such as headache, arthralgia, chills, fatigue, malaise and fever), and no difference in serious AE reported in our study. **Table 4** shows the unsolicited events that were collected and reviewed by the DSMB. Most of them were evaluated as non-severe, not related to study vaccines, and at the expected level for such population. Events that were hospital attended were included if deemed serious.

**Table 4:** Unsolicited adverse events per study arm.

Adverse Event	PCV13	Hib	TOTAL
Malaria	44	34	78
Pneumonia	14	5	19
Otitis media	2	6	8
Anaemia	1	7	8
Others	19	19	38
TB	3	2	5
TOTAL (p value = 0.20)	83	73	156

There was no statistically significant difference between study arms on hospital attendance for AEs per study arm as shown in **Table 5**.

**Table 5:** Hospital attendance for AEs per study arm.

Hospital attendance	PCV13	Hib	TOTAL
Yes	59	46	105
No	24	27	51
TOTAL (p value = 0.31)	83	73	156

The reported SAEs (**Table 6**) were expected in the study population and were considered not related to study vaccines. These SAEs were reported to the ethics committee, DSMB and TMDA.

**Table 6:** Serious adverse events per study arm.

SAE	PCV13	Hib	Total
Severe Malaria	1	4	5
Cryptococcal Meningitis	2	0	2
Severe Anaemia	0	1	1
Chicken pox	0	1	1
Septicaemia	1	0	1
Bacterial meningitis	0	1	1
Fatal SAE	3^*^	5^†^	8

*Causes of death: one each of severe malaria, Cryptococcal meningitis, and septicaemia

^†^Causes of death: severe anaemia in two cases; one each of bacterial meningitis, severe malaria and severe form of chickenpox.

## Discussion

The study revealed a non-significant reduction in the acquisition of new vaccine serotypes of *S. pneumoniae* in the recipients of PCV13 by nearly a third compared to those who received Hib vaccine (our primary question). However, if clearance of carriage was included in the evaluation, the study found evidence that PCV13 was effective in the combined effect of preventing new acquisition or clearing existing pneumococcal vaccine type carriage. It is also relevant to note a significant decline in the proportion of children carrying vaccine serotypes in the PCV13 arm by nearly 50% after receiving two doses of pneumococcal vaccine compared to pre-vaccination proportion.

We understand that vaccination prevents acquisition of carriage of vaccine serotypes; it does not terminate current carriage. This means there could be a chance that some children will receive the vaccination and become immunologically competent but this would not be detected by examining carriage as they may be carrying a vaccine serotype prior to vaccination. Some studies found that in similar populations turnover of nasopharyngeal carriage is about two months (24, 25). We believe that the rate of false negatives due to continuous carriage would be minimal.

The results of this trial have therefore shown a vaccine effect on overall carriage of vaccine serotypes of *S. pneumoniae.* Such vaccine effect was not observed for non-vaccine serotypes. The plausible explanation for these findings is probably related to the ability of HIV infected children to mount an adequate antibody response against the particular vaccine serotypes of *S. pneumoniae* and thereby both reduce carriage and interfere with acquisition of pharyngeal colonisation.

PCV is known to be directly protective in HIV positive children against pneumococcal disease (3, 26, 27). Our study suggests that PCV13 vaccine is effective in the combined endpoint of preventing acquisition of new vaccine serotypes and clearing existing carriage of vaccine serotypes. Therefore, PCV13 may be effective at least in the short-term (over about 6 months) in preventing IPD among HIV infected children by reducing nasopharyngeal carriage of *S. pneumoniae* which is a prerequisite for invasive pneumococcal disease to occur (10). If the vaccine is introduced to an extended age range beyond those currently covered by EPI schedule, there is likely to be an impact of PCV13 vaccines at community level as well. A study with pneumococcal conjugate vaccines in South Africa revealed similar findings with decline in vaccine serotypes and IPD among both HIV infected and uninfected children (28).

The immunogenicity results in our study revealed that HIV-positive children receiving PCV13 generally developed good immune responses to the majority of vaccine serotypes measured about 3 months post the second dose of vaccination. This is consistent with previous studies of seven- or nine-valent PCV in HIV-positive children, in which PCV was shown to be immunogenic (27, 29). However, in our study, the immune responses post 2 doses of PCV13 among HIV-positive children were suboptimal for some vaccine serotypes. Given that pre-vaccine levels were not considered when interpreting the lack of 4-fold rise in 7/13 serotypes; the data appear to show a lower magnitude of vaccine response among serotypes with higher pre-vaccine levels, which has been previously reported in studies of pneumococcal antibody response (13, 14, 16, 30–32), and of course, certain serotypes (e.g. serotype 3) are also known to be less immunogenic. This acknowledges the uncertainty of immunogenicity as a correlate for clinical protection and depending on the local epidemiology of circulating serotypes (32, 33), this finding adds some uncertainties in assessing the overall benefits of vaccinating HIV-positive children against pneumococcal disease using PCV13.

Our study also suggests a good safety profile of PCV13 among HIV-positive children, similar with the previous study assessing the use of PCV7 in children with immunodeficiency (26). In another study, HIV-positive children receiving PCV7 did not have significantly higher risk of adverse events than those who received control vaccine, although some particular adverse events were more frequent in the PCV7 vaccinees e.g. swelling and redness (26). To increase vaccine impact, we propose quicker identification of HIV-positive children aged ≥1 year in the community and vaccinate them with two doses of PCV13 if they have not had the vaccine. Policy makers should consider extending the coverage to protect HIV-infected children as they are at high risk of carrying pneumococci and also vulnerable to severe IPD and death.

We saw a rise of non-vaccine serotype colonisation making the overall pneumococcal isolation rate per visit similar. This suggests serotype replacement may occur at a relatively fast rate in this HIV infected population. It could also mean persistence of protection from acquisition of new pneumococcal serotypes by the vaccine is jeopardized by their disease condition, which is apparently evident in their immune responses to certain serotypes that were tested. As a result, some non-PCV13 serotypes may also cause IPD, perhaps this may explain an apparent increase of cases of pneumonia reported in the PCV13 arm compared to Hib arm. Although it should be noted that the diagnosis of pneumonia in the study setting, was based on clinical judgment according to IMCI classification where causes of pneumonia other than pneumococcus could not be ruled out.

We understand that our study findings may have some potential limitation such as: culture-based detection and the serotyping method used (Quellung reaction) can sometimes misidentify pneumococci or pneumococcal serotypes that require other methods for verification, but it still represent a reasonable approach, being regarded as a gold standard (34) despite its limitations (35). Also the putative antibody concentration of 0.35 ug/mL as set by the WHO (23) was determined based on protection from IPD in infants, but higher antibody levels may be required to prevent carriage and mucosal infections. Protective levels may also vary in older children and or adults.

The pneumococcal vaccine is vital for African countries like Tanzania where pneumococcal infections account for substantial mortality in young children. With the improved care for HIV-positive individuals leading to longer life expectancy, the anticipated benefit of rolling out safe, efficacious, and life-saving vaccines like PCV13 is becoming more profound. Our study suggests that the introduction of PCV13 targeting HIV-positive children in settings similar to Tanzania is likely to be associated with appreciable decrease in the acquisition and carriage of pneumococci, which is an important marker of the likely effect of the vaccine on pneumococcal disease.

## Data Availability Statement

The original contributions presented in the study are included in the article. Further inquiries can be directed to the corresponding author, and will be subject to approval from the collaborating institutions and signing the data transfer agreement (DTA) from the National Institute for Medical Research (NIMR), Tanzania.

## Ethics Statement

The study involving human participants was reviewed and approved by National Health Research Ethics Committee and the Tanzania Food and Drug Authority in Tanzania. Written informed consent to participate in this study was provided by the participants’ legal guardian/next of kin.

## Author Contributions

Conceptualisation (RB), Writing original draft (GMa), Data curation (GMa, JKY, VMB, AM, GMt), Formal analysis (GMa, JKY, GMt, RB, BA), Funding acquisition (RB), Investigation (GMa, GMt, AM, VMB, RB, LA, JM), Methodology (GMa, JKY, GMt, RB, BA, DS), Resources (RB, WK), Writing review and editing (GMa, JKY, GMt, VMB, AM, RB, BA, DS), Project administration (GMt, RB, WK). All authors contributed to the article and approved the submitted version.

## Funding

This was an investigator-initiated study sponsored by Pfizer to RB. The funder was not involved in the study design, collection, analysis, interpretation of data, the writing of this article or the decision to submit it for publication.

## Conflict of Interest

The study was funded by Pfizer, a company that also supplied the PCV13 vaccine. However, the company did not have any contribution on the analysis or interpretation of the study results.

RB received funding for an investigator-initiated study. JKY is currently a full time employee of Sanofi Pasteur; his contribution to this work was made before he joined the company.

The remaining authors declare that the research was conducted in the absence of any commercial or financial relationships that could be construed as a potential conflict of interest.
